# Research Progress in Fecal Microbiota Transplantation as Treatment for Irritable Bowel Syndrome

**DOI:** 10.1155/2019/9759138

**Published:** 2019-12-01

**Authors:** Yao Wang, Fengling Zheng, Shan Liu, Huanhuan Luo

**Affiliations:** ^1^School of Basic Medicine, Guangzhou University of Chinese Medicine, Guangzhou, Guangdong Province 510006, China; ^2^The Journal Center, Guangzhou University of Chinese Medicine, Guangzhou, Guangdong Province 510006, China

## Abstract

Irritable bowel syndrome is a functional disorder characterized by abdominal pain or discomfort associated with altered bowel habits. Due to the uncertainty of the pathogenesis of IBS and the diversity of its clinical manifestations, IBS cannot be completely cured. Increasing evidence suggests the key role of altered intestinal microbiota in the pathogenesis of IBS. Therefore, attention is being shifted to adjusting the changes in intestinal microbiota to control IBS symptoms. Fecal microbiota transplantation (FMT), antibiotics, probiotics, and synbiotics are currently often employed as treatment for IBS. And FMT is the most significant therapeutic efficacy with the least number of side effects. FMT provides a creative way to restore the abnormal gut microbiome in patients with IBS. But although current clinical studies confirm the effectiveness of FMT in the treatment of IBS, they are short-term studies of small samples, and there is still a lack of large-scale long-term studies. In this paper, we review the intestinal microbiota changes of IBS, the common methods of treating IBS with intestinal microbes, and the research status of FMT for the treatment of IBS. Finally, we put forward some opinions on the future research direction of FMT treatment of IBS.

## 1. Introduction

Approximately 100 trillion different microorganisms inhabit the human intestine [1]. The gastrointestinal tract harbors the largest microbial population in the human body, accounting for 80% of the total microbial biomass. Nearly 1,014 bacteria dominate the microbiota in the gastrointestinal tract, with the genome of the intestinal microbiota having been estimated to contain almost 100 times more genes than the human genome [2, 3]. In addition to bacteria, gut microbes include fungi, fields, viruses, and eukaryotes [4]. And the degree of parasitic infection may be related to differences in the gut microbiome [5]. The composition of the intestinal microbiota varies from one individual to another, and studies have shown that the intestinal microbiota plays a crucial role in human health and diseases via its involvement in the regulation of immune and metabolic functions [6–9]. Dysbiosis, defined as a microbial imbalance in the body [10], is an important cause of digestive tract diseases. Intestinal dysbiosis can lead to inflammatory bowel disease [11], chronic fatigue syndrome [12], obesity [13], cancer [14], metabolic diseases [15], and irritable bowel syndrome (IBS). In particular, IBS is a functional disorder characterized by abdominal pain or discomfort associated with altered bowel habits [16]. IBS diminishes patients' quality of life, resulting in psychological problems such as anxiety and depression [17]. Although IBS is not fatal [18], failure to treat IBS can lead to impairment in quality of life among IBS patients. Currently, IBS is mainly diagnosed based on medical history and physical signs, with the aid of endoscopic and imaging examinations to exclude organic diseases. According to the Rome IV diagnostic criteria for IBS [19], which were launched at the “Ninth Annual American Gastroenterological Association-Rome Foundation Lectureship” held at the 2016 Digestive Disease Week meeting, IBS can be categorized into the following four subtypes based on predominant bowel habits: IBS with predominant constipation (IBS-C), IBS with predominant diarrhea (IBS-D), IBS with mixed bowel habits (IBS-M), and unclassified IBS (IBS-U) [20]. Despite the availability of numerous drugs for the control of IBS symptoms, several patients do not respond well to these drugs [21]. The uncertainty about the pathogenesis of IBS and the diversity of its clinical manifestations present considerable difficulties for IBS treatment. An increasing body of evidence has confirmed that IBS is closely related to intestinal dysbiosis. Patients with different types of IBS develop diverse disorders associated with the microbiota and exhibit changes in the number and types of microbiota [22]. Furthermore, IBS patients have been reported to have a smaller number of intestinal *Bifidobacterium* species but a larger number of *Escherichia coli* [23]. The distal colonic mucosa in IBS-C patients contains fewer aerobic bacteria than that in healthy individuals [24]. IBS-D patients have a decreased number of *Bacteroides*, *Bifidobacterium*, and *Ruminococcus* species [25]. In comparison, IBS-M patients have a significantly decreased number of *Lactobacillus* species and an increased number of *Enterobacteriaceae* species in their feces and exhibit lower resistance to intestinal colonization than healthy individuals [26]. Lactulose hydrogen breath test can verify the occurrence of small intestinal bacterial overgrowth (SIBO) at varying degrees in IBS patients [25]. Therefore, attention is being shifted to adjusting the changes in intestinal microbiota in order to control IBS symptoms. Clinical research has begun to explore IBS treatment targeting the intestinal microbiota. Fecal microbiota transplantation (FMT), antibiotics, probiotics, and synbiotics [27] are currently often employed as treatment for IBS, with FMT showing the most significant therapeutic efficacy with the least number of side effects. FMT refers to the transplantation of a fecal matter solution from a donor into the gastrointestinal tract of a patient in order to directly change the recipient's microbial composition and hence treat the disease [28, 29]. An increasing number of clinical studies have investigated IBS treatment using FMT; nonetheless, there exist few reviews in this regard. Therefore, this paper is aimed at reviewing the progress and shortcomings in the use of FMT as treatment for IBS based on existing clinical studies so as to improve the application of FMT to IBS treatment.

## 2. Pathogenesis of IBS

IBS is a multifactorial disease with a complex pathogenesis and pathophysiological mechanisms. Current research suggests that the pathogenesis of IBS is related to the interaction between the brain-gut axis, immune system, and intestinal microecology [30]. PI-IBS patients exhibit increased peripheral blood levels of tumor necrosis factor-*α*, interleukin-1, and interleukin-6, with observable CD3 and CD25 cells and lymphocytes in the intestinal mucosa [31]. The underlying mechanisms responsible for PI-IBS remain unclear and may involve residual inflammation or persistent changes in mucosal immune cells, enterochromaffin-like cells, mast cells, intestinal nerves, and gastrointestinal microbes [32]. A number of research groups have attempted to evaluate the effects of the microbiome on the brain-gut axis. The brain-gut axis is a bidirectional communication pathway deemed essential for homeostasis maintenance, and part of the brain-gut axis causes important pathophysiological effects in regulatory disorders; that is, changes in brain-gut interactions have a certain effect on intestinal inflammation and abdominal pain symptoms [33]. The pathogenesis of IBS cannot be explained by a single mechanism, and changes in the intestinal flora have become the focus of concern [34].

## 3. Intestinal Microbiota and IBS

Microorganisms help maintain the human body's normal physiological functions [35, 36]. The intestine harbors approximately 1,150 species [2], which mainly include anaerobic bacteria such as *Bacteroides*, *Bifidobacterium*, *Actinomyces*, *Firmicutes*, and *Proteobacteria* species. The genome of the intestinal microbiota contains almost 100 times more genes than the human genome [3]. It is generally believed that the pathogenesis of IBS is related to the intestinal barrier function, central and splanchnic neurological changes, mental and psychological stress, microinflammation, and intestinal dysmotility [4]. Recent studies have reported that intestinal dysbiosis in IBS patients may be the initiating factor in the pathogenesis of IBS [37]. The human intestinal microbiota is an extremely complex and metabolically active microbial ecosystem [34] involved in the development of the host's immune system [38, 39], maintenance of normal physiological functions of the digestive system [40], and fermentation of undigested carbohydrates [41]. The intestinal microbiota comprises three types of bacteria—namely, symbiotic bacteria, conditionally pathogenic bacteria, and transient bacteria. The classic findings to confirm the bacteria in IBS might be using rifaximin in IBS-D by RCT studies [42]. Symbiotic bacteria are important for healthy digestion in that they produce enzymes and metabolites that help the body absorb essential nutrients and vitamins [43]. Conditionally, pathogenic bacteria are invasive under certain conditions, whereas transient bacteria can cause disease when dysbiosis occurs and their number exceeds the normal level. The physiological functions of the intestinal microbiota can be primarily summarized into the following five aspects: (1) regulation of mucosal immune responses in the gastrointestinal tract; (2) activation of immune factors, strengthening of immune functions, and enhancement of disease resistance; (3) promotion of immune organs' growth and development and initiation of immune response; (4) creation of a bacterial barrier; and (5) interference with cellular immunity and stimulating the body to produce an immune response when existing as probiotics. Under normal circumstances, the intestinal microbiota can maintain a dynamic balance to promote bodily health. However, intestinal dysbiosis can induce or aggravate diseases [44] such as metabolic syndrome, IBS, and inflammatory bowel disease and is associated with some extraintestinal diseases [45, 46]. In addition, there are some studies on fungi, parasites, and viruses in intestinal microbes that are also involved in the development of IBS. In addition, some studies have shown that fungi, parasites, and viruses in gut microbes are also associated with the occurrence of IBS. For example, *S. boulardii* can reduce the number of intestinal peristalsis in patients with irritable bowel syndrome and diarrhea (IBS-D) [47]. A case-control control study has shown the infection rate of intestinal parasites in patients with IBS which is not high compared to healthy people [48]. Nourrisson et al. showed that the level of intestinal *Bifidobacterium sp.* in male IBS-C patients was lower than in the normal control group. No studies have clarified which specific microbes are particularly relevant to IBS [49], and healthy populations and patients with severe or mild/moderate IBS can be identified based on the structural features of the intestinal microbiota [6]. Microbial diversity in fecal samples from IBS-D patients is significantly reduced [50], with the intestinal microbiota mainly characterized by an increased number of *Enterobacteriaceae* species and a decreased number of *Lactobacillus*, *Bifidobacterium*, and *Bacteroides* species [51]. The intestinal microecology in IBS-C patients is characterized by an increased number of *Firmicutes* and *Bacteroidetes* species [52], with constipation resulting from excessive methane production [53]. In comparison, IBS-M patients show a significantly increased number of *Enterobacteriaceae* species in their feces, significantly decreased number of *Lactobacillus* species, reduced resistance to intestinal colonization, and increased abundance of *Bacteroidetes* species [53, 54]. Changes in the intestinal microbiota in IBS-U patients remain unclear.

The severity of IBS symptoms is inversely correlated with microbial richness and unique microbial markers [55]. For instance, *Bifidobacteria* are inversely associated with pain [56], and both *Bifidobacteria* and *Lactobacilli* are inversely associated with defecation frequency [53]. In addition, cyanobacteria are associated with satiety, bloating, and gastrointestinal symptoms, whereas proteobacteria are associated with psychological and pain thresholds [57]. *Ruminococcus* species are associated with the severity of IBS symptoms [58]. IBS patients mainly show a decrease in the number of dominant intestinal microbiota, intestinal microbial diversity, stability of intestinal microecology, and an abundance of *Bifidobacteria* and *Lactobacilli*, along with an increase in the number of enterobacteria and a decline in resistance to intestinal colonization. Patients with different types of IBS have diverse changes in the intestinal microbiota. In clinics, it is possible to conduct targeted treatment according to changes in the intestinal microbiota. Promoting intestinal microbial diversity and restoring the balance of the intestinal microbiota are particularly important in IBS treatment.

## 4. Application of Interventions Targeting the Intestinal Microbiota to IBS Treatment

Currently, the use of fermentable oligosaccharides, disaccharides, monosaccharides, and polyols (FODMAP), antibiotics, and probiotics as treatment for IBS has achieved a certain level of therapeutic efficacy. Commonly found in food, FODMAP are short-chain carbohydrates that are not readily absorbed in the small intestine. A low-FODMAP diet for 3 weeks has been shown to significantly improve IBS symptoms, increase the abundance and diversity of *Actinomyces* in the fecal microbiota, and decrease the urinary concentration of histamine [59]. It is currently accepted that SIBO is most closely related to IBS, prompting the proposal that antibiotics could be used as treatment for IBS [60]. Pistiki et al. [61] isolated 170 types of aerobic bacteria from the intestines of patients diagnosed with SIBO-induced IBS.

Furthermore, they reported that the in vitro use of 32 *μ*g/mL rifaximin inhibited 85.4% of *E. coli*, 43.6% of *Klebsiella* species, 34.8% of *Enterobacteriaceae* species, 54.5% of other *Enterobacteriaceae* species, 82.6% of Gram-negative non-*Enterobacteriaceae* species, and 100% of *Enterococcu*s faecalis and *Staphylococcus aureus*, suggesting the antibacterial effect of rifaximin on small intestinal bacteria related to SIBO. In addition, patients with nonconstipation IBS showed improvement in bloating, abdominal pain, and fecal traits after treatment with rifaximin [62], and 5-HT3 receptor antagonists can improve symptoms in IBS-D patients [63]. The mechanism by which antibiotics treat IBS may be related to the changes in the ratio of intestinal microbiota and prevention of intestinal inflammation and visceral hypersensitivity due to stress [64].

Probiotics are living microorganisms that are beneficial to the human body [65]. Probiotics can restore the ecological balance of the intestinal microbiota and alter the expression and redistribution of tight junction proteins, can restrict the absorption of harmful molecules in the intestinal lumen by reducing intestinal mucosal permeability, and can affect the inflammatory response of epithelial cells to the stimuli in the intestinal lumen by secreting various factors, thereby reducing mucosal inflammation while acquiring immunity by affecting mucosal T-cells in the lamina propria [66, 67]. Probiotics can improve abdominal pain symptoms in IBS patients and alleviate overall symptoms but are not effective in improving the symptoms of bloating, constipation, and diarrhea [68]. Supplementation with *Lactobacilli* or *Bifidobacteria* not only reduces the intestinal mucosal inflammatory response in PI-IBS patients [69] but also significantly improves the symptoms of diarrhea and constipation [70]. A study by Johnsen et al. showed that FMT could significantly reduce the symptoms of IBS patients but did not affect the patients' microbiome [71]. According to a 2018 study, the microbiome of IBS patients was closely similar to that of donors after FMT capsule treatment, and FMT capsules had a significant effect on the recipients' microbiome [72]. Methods for FMT include the use of nasogastric or nasojejunal tube and endoscopic approaches, with the colonoscopic approach showing the best performance. The recent meta-analysis shows FMT treatment CDI (Clostridium difficile infection) has a good effect [73].

## 5. Current Status of FMT

### 5.1. Donor Selection

As is the case with other treatments, FMT poses some risk. Despite the absence of a uniform standard for “qualified” microbial communities, careful selection of FMT donors is necessary. Only a healthy individual without autoimmune or metabolic disease, family history of malignant disease, or any potential pathogen can become a fecal donor [74]. Although patients are allowed to choose relatives as the source of transplanted feces in early FMT [75, 76], there exist no definitive statistical data confirming that selecting relatives as donors would improve the quality of FMT. Irrespective of the type of donor selected for FMT, each donor should be carefully and thoroughly screened prior to FMT [77–79]. Different patients and donors are subject to individualized adjustments and require additional examinations [80]. Currently, the prevailing screening method is to ensure—as far as possible—the absence of diseases due to intestinal microbes, blood-related disorders, or bacterial or parasitic infections transmitted through FMT while fulfilling the inclusion criteria.

### 5.2. Methods for the Preparation of Mixed Liquid

Currently, the main preparation method involves repeatedly weighing, preparing, filtering, and centrifuging the fecal liquid in order to produce a mixture of fecal bacteria. This conventional preparation method is time-consuming, labor-intensive, and inefficient, failing to meet clinical requirements. Cui et al. [81, 82] developed a smart microbiota treatment system that standardizes the procedure of precise fecal bacterial processing, multistage filtration, repeated centrifugation, concentration, and enrichment. Because of rapid preparation, which minimizes air contact, a large number of anaerobic bacteria in the fecal liquid are allowed to survive to the greatest extent, thus making the prepared fecal microbiota basically similar to the original fecal microbiota with respect to structural composition. Studies have shown that suspension prepared with water has a higher resolution than that prepared with physiological saline, although the recurrence rate of the former is higher; in addition, yogurt, milk, or psyllium-containing brine [83] can be used as a diluent. Nonetheless, some precautions must be taken: (1) In order to avoid mass reproduction of aerobic bacteria, the preparation process should be simple as much as possible [84]. (2) After processing, the mixture can be directly injected into the gastrointestinal tract or can be prepared as capsules for administration [79]. (3) Lyophilized capsules are small in volume and can be stored for a long time [85]. (4) A case report in 2012 indicated that the use of standardized frozen feces was not statistically different from the use of fresh feces [86].

### 5.3. Route of Administration for FMT

The fecal liquid can be injected into the intestine through a variety of ways, mainly via nasogastric tubes, nasojejunal tubes, upper gastrointestinal endoscopes, colonoscopes, or retention enema. Colonoscopy enables the lesion to be directly observed; however, being an invasive examination, colonoscopy may cause damage to the colon and is costly [84]. Although the use of enema is less costly and invasive than the colonoscopic approach, its performance is less satisfactory owing to the limited range of intestinal sites that the enema can reach [84]. The fecal liquid can also be injected via the upper digestive tract, which is simple and has few complications. Only a limited number of studies have explored the effect of administration route for FMT on clinical efficacy, and a large number of clinical trials are required for verification. The optimal administration route may depend on the location of the lesion, characteristics of the disease, and general condition of the patient. No consensus on the frequency of FMT has been achieved yet, and FMT may be performed either multiple times (intermittent or continuous administration) or as a single administration [79]. Each route has its own advantages and disadvantages, and the appropriate route should be selected based on the characteristics of the disease. For instance, in the case of abdominal diseases related to the small intestine, FMT is recommended to be performed via the duodenum. Crohn's disease often affects the entire gastrointestinal tract, preferentially the distal ileum and right hemicolon. Therefore, the upper gastrointestinal route may be more appropriate. Using the colonoscopic route in patients with severe colonic inflammation or colonic flatulence may entail a high risk; hence, the upper gastrointestinal route can be adopted instead [87]. The administration route should be determined according to the specific conditions of the patient.

## 6. Clinical Efficacy of FMT in Treating IBS

Probiotics can restore the intestinal microecology in IBS patients. Dissimilar to probiotics containing a small number of bacterial strains, FMT feces includes almost all bacteria from healthy donors. To some extent, fecal bacteria are the ultimate probiotics in humans [88]. In 1989, McEvoy [89] was the first to use FMT as treatment for IBS and inflammatory bowel disease, with a cure rate of 36% (20/55). Pinn et al. [90] employed FMT to treat refractory IBS patients (9 IBS-D patients, 3 IBS-C patients, and 1 IBS-M patient) and reported an overall remission rate of 70% for IBS, including improvement in abdominal pain (72%), bowel habits (69%), indigestion (67%), bloating (50%), and venting (42%). Moreover, Mazzawi et al. [91] explored the effects of FMT on symptoms and duodenal enteroendocrine cells in IBS patients and reported the effectiveness of FMT in treating IBS-D patients, revealing that IBS symptom scores for abdominal pain (*P* = 0.005), diarrhea (*P* = 0.004), and anorexia (*P* = 0.096); IBS severity scores (*P* = 0.0002); and Bristol Stool Scale scores (*P* = 0.02) were significantly decreased after FMT treatment for 3 weeks. Gaidar et al. [92] investigated the effectiveness of colonoscopy-assisted FMT in treating refractory IBS-D, IBS-C, and IBS-M patients. They reported that 75% of patients experienced relief from abdominal pain (*P* < 0.01) and that stool frequency and consistency in all IBS-M and IBS-D patients returned to normal. In 2017, the first randomized controlled trial (RCT) on FMT treatment for IBS [93] was conducted in Norway, primarily enrolling severe IBS patients who met the Rome III criteria. The patients were assigned to a group (*n* = 60) comprising subjects who received 50–80 g of fresh FMT (used on the same day) or frozen FMT and a group (*n* = 30) consisting of subjects who received his or her own feces as placebo. Transplantation was performed with colonoscopy. After 3 months of FMT treatment, the Irritable Bowel Syndrome Symptom Severity Scale scores decreased by more than 75 points in 36 out of 55 subjects who were actively treated (65%) and 12 out of 28 subjects who received placebo (43%) (*P* = 0.049), indicating that therapeutic efficacy was significantly better in the treatment group than in the placebo group. In 2018, Holvoet et al. [94] performed an RCT that included patients who were diagnosed with Rome III IBS complicated with severe bloating but did not have constipation. A total of 64 patients were allocated to the fresh FMT group, with each subject treated with either frozen feces from two donors or the subject's own feces. Transplantation was performed using an electromagnetically guided nasojejunal tube. At week 12, 49 FMT recipients showed adequate relief from IBS symptoms and bloating, compared with 29 in the control group (*P* = 0.004). In a recent RCT conducted by Johnsen et al., the IBS severity score of patients who received donor FMT via colonoscopy decreased, as compared with that of patients who received autologous FMT [71]. Nevertheless, this RCT showed that the effect of autogenous feces transported via colonoscopy or nasal intestinal tubes was better than that of donor feces [71, 95]. Some studies reported that FMT has no significant effect on IBS treatment. The results of the RCT indicated that when FMT was administered via capsules, placebo capsules were better than capsules containing donor feces [72, 96, 97]. The placebo effect is common in clinical trials on IBS [98], which may be related to IBS itself being a functional bowel disease [99]. Thus far, trials on FMT-based treatment for IBS have been mostly conducted with small sample sizes and lack long-term follow-up. The relationship between gut microbes and IBS remains not entirely clear, which may be the reason for the ineffective FMT treatment. Furthermore, the relationship between intestinal microbes and IBS is not fully understood, which may explain why FMT is not effective in treating some IBS patients. Adverse reactions to FMT treatment should also attract our attention; most of these adverse reactions are gastrointestinal symptoms, most patients experience transient diarrhea after FMT treatment, and a few may manifest symptoms such as bloating and belching, which usually disappear after 2–3 days [85]. Additionally, studies reported that adverse reactions, including death (0.25%), perforation/tear (0.25%), and Gram-negative bacteremia (0.34%), might occur in CDI patients receiving FMT; although the incidence is not high, we should consider it important because of the threat that adverse reactions constitute to health [100]. Owing to a short follow-up period, it is not possible to assess long-term efficacy or the occurrence of an underlying disease. Therefore, high-quality RCTs with large samples and long-term follow-up are required to further investigate the therapeutic effect of FMT on IBS.

## 7. Conclusions

Although FMT for CDI has a significant effect, its effectiveness in treating IBS needs to be further explored. Reconstructing a healthy microecology is desirable, but feces contain not only bacteria but also viruses, phages, fungi, protozoa, cells, mucus, enzymes, and metabolites—all of which affect the entire human ecosystem. In future research, the underlying mechanism of microbiome-host interaction and the role of microbiome in the pathogenesis of the disease should be elucidated. To this end, it is necessary to explore the specificity of the intestinal microbiota in each IBS subtype (i.e., specifying which bacteria are associated with which symptoms); by doing so, we may identify a different treatment approach. Moreover, exploring whether FMT restores a patient's intestinal microecology or the functions of some microbes is recommended. It is possible to further clarify the changes in the structural composition of the intestinal microbiota and the difference in intestinal microbiota between the patient and the donor before and after FMT treatment by employing metagenomic detection technology.
